# Protocol for Short- and Longer-term Spatial Learning and Memory in Mice

**DOI:** 10.3389/fnbeh.2017.00197

**Published:** 2017-10-17

**Authors:** Emily F. Willis, Perry F. Bartlett, Jana Vukovic

**Affiliations:** ^1^School of Biomedical Sciences, University of Queensland, Brisbane, QLD, Australia; ^2^Queensland Brain Institute, University of Queensland, Brisbane, QLD, Australia

**Keywords:** cognition, hippocampus, neurogenesis, acquisition, active place avoidance, learning

## Abstract

Studies on the role of the hippocampus in higher cognitive functions such as spatial learning and memory in rodents are reliant upon robust and objective behavioral tests. This protocol describes one such test—the active place avoidance (APA) task. This behavioral task involves the mouse continuously integrating visual cues to orientate itself within a rotating arena in order to actively avoid a shock zone, the location of which remains constant relative to the room. This protocol details the step-by-step procedures for a novel paradigm of the hippocampal-dependent APA task, measuring acquisition of spatial learning during a single 20-min trial (i.e., short-term memory), with spatial memory encoding and retrieval (i.e., long-term memory) assessed by trials conducted over consecutive days. Using the APA task, cognitive flexibility can be assessed using the reversal learning paradigm, as this increases the cognitive load required for efficient performance in the task. In addition to a detailed experimental protocol, this paper also describes the range of its possible applications, the expected key results, as well as the analytical methods to assess the data, and the pitfalls/troubleshooting measures. The protocol described herein is highly robust and produces replicable results, thus presenting an important paradigm that enables the assessment of subtle short-term changes in spatial learning and memory, such as those observed for many experimental interventions.

## Introduction

Over the past few decades, the development of behavioral tasks assessing spatial learning and memory in rodents has furthered our understanding of the role of the hippocampus in such cognitive functions (Cimadevilla et al., 2000a; Bannerman et al., 2004). One such behavioral task is the active place avoidance (APA) task, which assesses hippocampal-dependent spatial learning and memory (Cimadevilla et al., 2000b, 2001a; Vukovic et al., 2013; Leinenga and Gotz, 2015; Cleland et al., 2017). The APA task requires a mouse to continuously integrate visual cues to orientate itself relative to the room in order to actively avoid a stable shock zone within a rotating arena. This is termed allothetic navigation, where the animal uses distant landmarks and their relationships to avoid the shock zone (Cimadevilla et al., 2001a). The spatial learning and memory assessed by the APA task is hippocampal-dependent, and is not a stress response, with the foot shocks delivered upon entry into the shock zone demonstrated not to influence corticosterone levels (Lesburgueres et al., 2016). During this task, mice rapidly and robustly learn the location of the shock zone over a single 20 min learning event, with long-term spatial memory encoding and retrieval able to be examined by testing the mice in several APA trials conducted over consecutive days.

Herein, we describe the step-by-step procedures of the APA protocol, which, in brief, consists of (1) habituation, (2) 1- and 5-day learning paradigms, (3) a “white barrel” training session, and (4) reversal learning. Before testing, mice are handled once daily for 2 weeks to minimize handling-related stress that could impair performance in the task. Mice are first habituated to the testing arena, during which time they can freely explore. One day later, mice are tested in a 20-min APA task trial, during which time the mice are observed to develop specific place avoidance, with significantly fewer entries into the shock zone during the last 5 min of the trial compared to the first 5 min. Unlike most cognitive tests assessing hippocampal-dependent learning and memory, the APA task thus allows for the examination of rapid spatial learning acquisition (i.e., short-term memory, developing within a few seconds or minutes) during a single testing event.

The APA task can also be used to assess encoding and retrieval of spatial memory (i.e., long-term memory, lasting for at least 24 h). In this protocol, mice are tested over five consecutive days, with each trial 24 h apart. Mice are typically observed to enter the shock zone significantly less often on days 4 and 5 of testing, as compared with day 1, with fewer shocks received and an increased latency to first entry into the shock zone. This spatial learning and memory is proven allothetic, as removal of visual cues impairs performance in the task. Lastly, this specific place avoidance can be reversibly learnt, with young wild-type mice shown to rapidly learn the location of a new shock zone, highlighting the cognitive flexibility of the mice.

## Materials and equipment

All materials and equipment used in this protocol are listed in Table 1.

**Table 1:** Materials and equipment.

**Name of equipment/material**	**Company**	**Catalogue/model number**
APA arena with grid floor (diameter 77 cm) fenced with Perspex clear circular boundary (32-cm high)	Bio-Signal Group, NY, USA	N/A
20-cm diameter clear Perspex cylinder	Made in-house	N/A
Digital video camera	Point Gray, USA	FL2-03S2M-C
Tracker software	Bio-Signal Group, NY, USA	Version 2.36
Track Analysis software	Bio-Signal Group, NY, USA	Version 2.2
70% ethanol	N/A	N/A
GraphPad Prism	GraphPad Software, CA, USA	Version 7.02

### Ethical statement

All experiments were conducted in accordance with the Australian Code of Practice for the Care and Use of Animals for Scientific Purposes, with local approval from The University of Queensland's Animal Ethics Committee. Twelve 3-month-old female C57BL/6 mice were used in this study, housed in groups of four mice per cage and maintained on a 12-h light dark cycle with food and water provided *ad libitum*. In the (unlikely) event that a mouse receives 15 shocks within a 5 min interval during the 20 min trial, or if the mouse appears overly stressed during testing as evident by excessive vocalization, excessive aggressive behavior toward the experimenter, or excessive jumping behavior, then testing should be terminated for ethical reasons.

### Stepwise procedures

#### Behavioral apparatus

**Figure 1:**
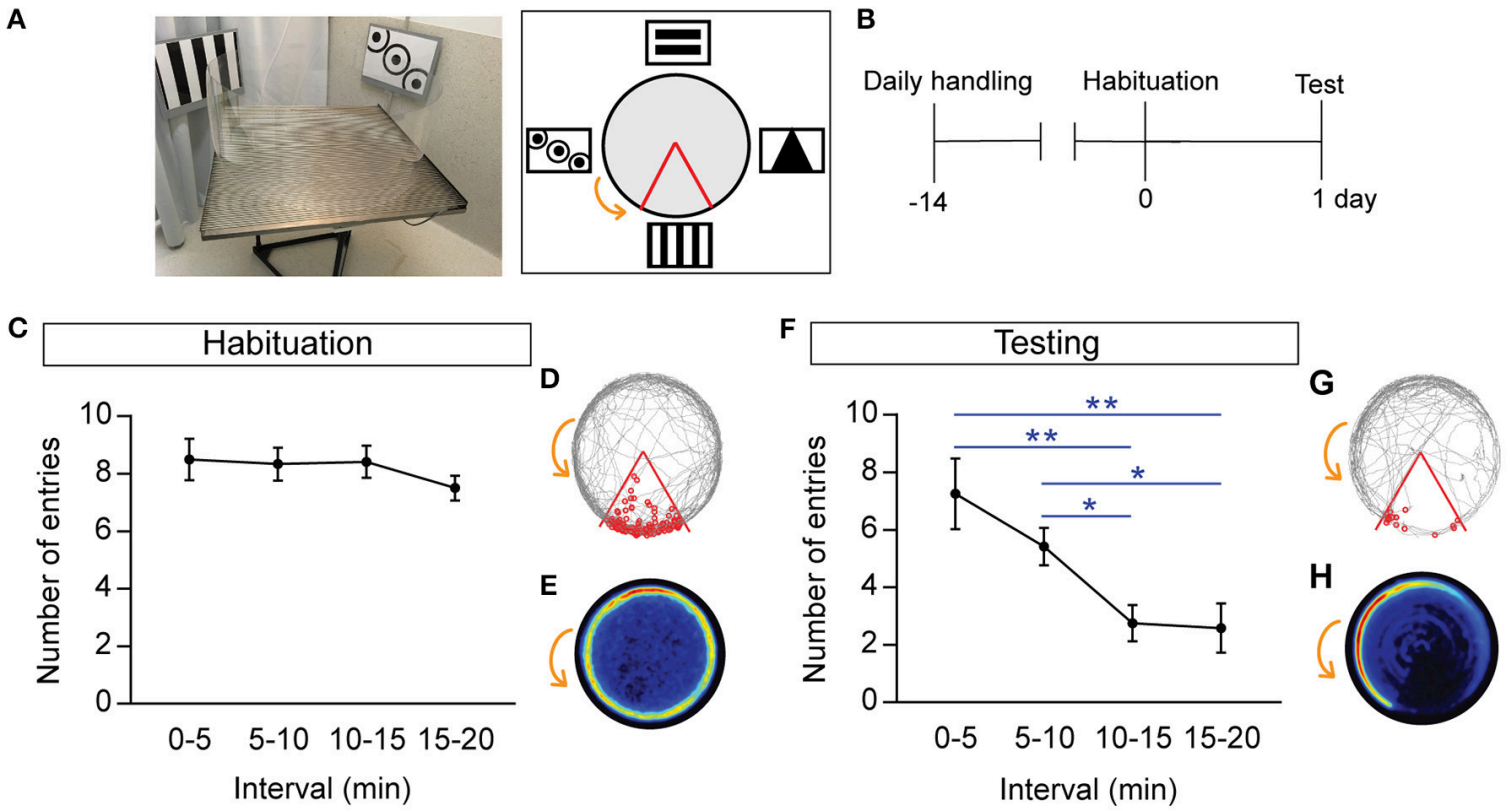
One-day learning paradigm demonstrating rapid acquisition of spatial learning during the active place avoidance task. **(A)** Photo (left) and schematic (right) of the testing arena and room. The large black and white extra-maze visual cues and shock zone location (red boundary) are depicted, with the counter-clockwise rotation of the arena indicated by the orange arrow. **(B)** Schematic of experimental timeline involving 14 days of handling followed by habituation (day 0), and testing (day 1). **(C,F)** Number of entries into the shock zone during habituation **(C)**, and trial **(F)**. **(D,G)** Representative track during habituation **(D)**, and trial **(G)**, where the gray line represents the path of a single mouse and each entry into the shock zone (red boundary) is visualized by a small red circle. **(E,H)** Heat maps during habituation **(E)**, and trial **(H)**, showing mice developing place-specific avoidance during the trial. Heat maps represent the merged maps of all mice, where the color of a pixel represents the average trajectory of tracks at that location (blue = low, red = high proportion of time; *n* = 12). All data is represented as mean ± SEM; ^*^P < 0.05, ^**^P < 0.01, *n* = 12.

1.1 The APA apparatus (Bio-Signal Group) consists of an elevated arena with a metal grid floor (diameter 77 cm) fenced by a 32 cm-high transparent circular boundary.
  1.1.1. The spacing of bars composing the metal grid are 0.5 cm apart, with bars having a diameter of 0.3 cm.
1.2 The arena is placed directly under a fitted camera, with a gap of at least 30 cm between and the arena and room walls.
1.3 Lighting (at the center of the arena), as measured using the light meter, is set at 70% white light.
1.4 Visual cues are placed on the room walls: four different cues are placed on four different walls. The visual cues are large black and white symbols/shapes, which are A3 in size (297 mm width × 420 mm height; Figure 1A). Other extra-maze visual room cues are controlled for by the white room walls and a gray curtain screening the experimenter from view.
1.5 The arena rotates counter-clockwise (1 rpm) and an electric shock can be delivered through the grid floor, which also rotates. The location of the shock zone (90° angle, width: 60°, inner radius: 0, outer radius, 127.50, i.e., whole quadrant from wall to center) remains constant (i.e., does not rotate) in relation to the room's coordinates. Rotation of the arena carries the mouse into the shock zone unless the animal actively avoids the area.
  1.5.1 The shock is delivered by the constant current source output, distributed across the metal grid by the Shock Scrambler (BioSignal Group Corp), which rapidly switches (“scrambles”) the shock signal, delivering the shock via the grid cable across the various metal bars defined as the shock zone. The shock is evenly distributed across the various metal bars.
1.6 Entrance into the shock zone leads to the delivery of a brief foot shock (500 ms, 60 Hz, 0.5 mA). If the mouse remains in the shock zone after the initial shock, further shocks are delivered at 1.5-s intervals until the mouse moves out of the zone.
1.7 The position of the mouse in the arena, relative to the shock zone, is tracked using the overhead camera linked to Tracker software (Bio-Signal Group, version 2.36). The center-point of the mouse is tracked. The position of the shock zone is defined by the arena room frame. Entry into the shock zone is defined by the center-point of the mouse entering the shock zone, as tracked by the overhead camera.
1.8 During the trials, the experimenter sits quietly behind a screening curtain, where the computer and software system is also housed. A video link from the camera to the computer allows the experimenter to watch the arena and intervene if required.
1.9 The recorded tracks for each mouse are analyzed using Track Analysis software (Bio-Signal Group, version 2.2).
1.10 To eliminate any odor cues that might impair performance in the task, the metal grid, underlying floor, and walls of the arena are cleaned with 70% ethanol before each trial. This includes removing any scat or urine from the arena at the end of each trial.
  1.10.1 Throughout behavioral testing, the experimenter should use the same laboratory coat. This limits olfactory cues that may stress the mouse and impair performance in the task.
1.11 The animals to be tested (kept in their home cages) should be moved into the testing room at least 30 min before the start of the trial. The lid of the home cages should be closed during the trial, again to limit olfactory cues.
1.12 During the trial, while the animal is in the arena performing the task, any noise should be limited, as this will give the animal another cue, affecting performance in the task.
1.13 The bedding of the animal's home cage should not be changed on the day of and/or throughout behavioral testing as this may provide a new stimulation, and could affect performance.

### Testing procedures

#### Habituation

2.1 All testing should be conducted during the light cycle of the mice, at approximately the same time each day.
2.2 To minimize handling-related stress, each mouse is handled daily, for 30 s–1 min, for the 2 weeks prior to testing, by the experimenter who will be conducting the task; ideally, at the same time each day Figure 1B). Handling involves the experimenter picking up the mouse (by the tail) from their home cage, and placing on the back of her/his gloved hand. The mouse is then returned to its home cage.
2.3 Twenty-four hours after the last daily handling, mice are habituated to the training room. This consists of moving the mice (in their home cage) to the training room and handling each mouse (as described above) for 30 s^−1^ min before returning it to its home cage.
2.4 The day after training-room habituation (step 2.2), mice are habituated to the testing arena by being placed in the counter-clockwise rotating arena (1 rpm), with the shock turned off, and allowed to explore the arena for 20 min.
2.5 The number of entries into the future shock zone, and the distance traveled during 5-min intervals within the 20-min trial are recorded.

#### 1-day learning paradigm

3.1 At the start of a trial, mice are transferred from their home cage to the testing arena using the method of transfer described in step 2.2.
3.2 The mouse's starting position within the arena is always near the Perspex wall, facing the wall opposite the shock zone.
3.3 Mice are tested over one 20-min training session.
3.4 Following completion of the trial, the mouse is transferred back to its home cage.
  3.4.1 After completion of the trial, if the mouse is overly stressed, a clear Perspex cylinder (smaller in diameter than that which surrounds the arena) may be placed over the mouse, in order to pick up and move the mouse back to its home cage more easily.
3.5 The extent of spatial learning and memory is assessed by measuring the following parameters: number of entries into the shock zone during 5-min intervals within the 20-min trial, number of shocks received during 5-min intervals within the 20-min trial, and latency of first entry into the shock zone.
  3.5.1 The number of entries and shocks received is used as a measure of cumulative performance in the task. The latency to first entry is used as a measure of in- or between- (see below) session learning and memory (i.e., retrieval of memory from the previous trial).
3.6 The total distance traveled during 5-min intervals within the 20-min trial is also measured.

#### 5-day learning paradigm

4.1 Twenty-four hours after habituation (section Habituation), mice are tested over five 20-min trials, with each trial 24 h apart. The first of the five trials begins 24 h after habituation in the arena (i.e., the 5-day learning paradigm includes the trial conducted for the 1-day learning paradigm).
4.2 During each trial, the location of the shock zone and the visual cues remain constant.
4.3 The extent of spatial learning and memory over the trial period is assessed, as mentioned in step 3.5.

#### White barrel training session

5.1 Twenty-four hours after the last trial, all visual cues are removed: the clear arena boundary is replaced with a white opaque boundary (“white barrel”) and the extra-maze visual cues are removed from the testing room walls. This allows for the determination of spatial learning in the absence of visual cues, and confirms the animals are orientating themselves using the allothetic room cues.
5.2 Spatial learning is assessed using the parameters mentioned previously (see step 3.5).

#### Reversal learning

6.1 Twenty-four hours after the “white barrel” training session, mice are tested in a reversal learning paradigm.
6.2 During reversal learning, the location of the shock zone is reversed (i.e., 180° opposite to the original location) with the same spatial room cues consisting of black and white symbols/shapes as those used for habituation, and the 1- and 5-day learning paradigms.
6.3 Mice are tested, as described above in section 1-day Learning Paradigm for the training session, with reversal learning assessed in five 20-min trials, each 24 h apart.
6.4 Again, spatial learning is assessed using parameters mentioned in step 3.5.

#### Data analysis

7.1 For habituation, the number of entries and distance traveled during 5-min intervals within the 20-min trial are analyzed using a repeat-measures one-way ANOVA with a Bonferroni *post-hoc* test using GraphPad Prism software (version 7.02). Differences are considered significant when *P* < 0.05. Number of entries and distance traveled are presented as mean ± SEM.
7.2 For the 1-day paradigm and white barrel trial, the number of entries into the shock zone, number of shocks received, and distance traveled during 5-min intervals within the 20-min trial are analyzed using a repeat-measures one-way ANOVA with a Bonferroni *post-hoc* test and Geisser-Greenhouse correction using GraphPad Prism software (version 7.02). Differences are considered significant when *P* < 0.05. Number of entries and distance traveled are presented as mean ± SEM.
7.3 For the 5-day paradigm, statistical differences between the total number of entries, number of shocks received, and distance traveled during the 20-min trial are analyzed using a repeat-measures one-way ANOVA, with Bonferroni *post-hoc* test and Geisser-Greenhouse correction. Spatial memory is assessed by latency to first entry into the shock zone (seconds) during days 1–5 of testing, with differences analyzed using a repeat-measures one-way ANOVA with a Bonferoni *post-hoc* test. Differences are considered significant when *P* < 0.05.
7.4 For reversal learning, spatial learning is assessed in the same manner as the 5-day paradigm.

## Anticipated results

### Acquisition of spatial learning during a single learning event

During habituation, when the mice explored the testing arena, there was no significant difference in the number of entries (Figure 1C), or the distance traveled, during any of the time intervals (entries: 0–5 vs. 15–20 min: 8.5 ± 0.72 vs. 7.5 ± 0.44, respectively; distance: 0–5 vs. 15–20 min: 18.34 ± 1.01 vs. 16 ± 0.544 m, respectively). The representative track (Figure 1D) and the merged heat maps (Figure 1E; all heat maps represent the merged average of *n* = 12 mice) demonstrate that all animals actively explored the testing arena, and typically stay close to the boundary during habituation.

During the APA trial, spatial learning was assessed throughout the 20-min trial, with mice entering the shock zone significantly less often in the 10–15 min and 15–20 min intervals, compared with the 0–5-min interval (0–5 vs. 10–15 min: *P* = 0.0053; 0–5 vs. 15–20 min: *P* = 0.013; 5–10 vs. 10–15 min: *P* = 0.019; *n* = 12; Figure 1F). The number of entries closely reflected the number of shocks received, with mice that quickly escaped the shock zone receiving significantly fewer shocks in the 10–15 and 15–20-min intervals compared with the 0–5-min interval (0–5 min: 8.58 ± 1.56 vs. 10–15 min: 3 ± 0.69, *P* = 0.0076; 0–5 min: 8.58 ± 1.56 vs. 15–20 min: 2.75 ± 0.89, *P* = 0.013). This suggests that mice rapidly learn the location of the shock zone and develop the ability for place-specific avoidance. During the APA trial, the mice developed a task-specific place avoidance of the shock zone, as evidenced by the representative gray tracked path opposite to the shock zone (Figure 1G), and their spatial learning was also reflected in the merged heat map showing the highest intensity 45° counter-rotation from the shock zone (Figure 1H). There was no difference between distance traveled during the first 0–5 min interval compared with the last 15–20 min interval (0–5 min: 13.77 ± 0.35 m vs. 15–20 min: 12.82 ± 0.35 m, *P* = 0.17; *n* = 12). There was a moderate association between the number of entries and the distance traveled, with fewer entries associated with greater distance traveled (*R*^2^ = 0.41, *P* = 0.025, *n* = 12).

### Spatial learning and memory in APA task is allothetic and reversibly learnt

**Figure 2:**
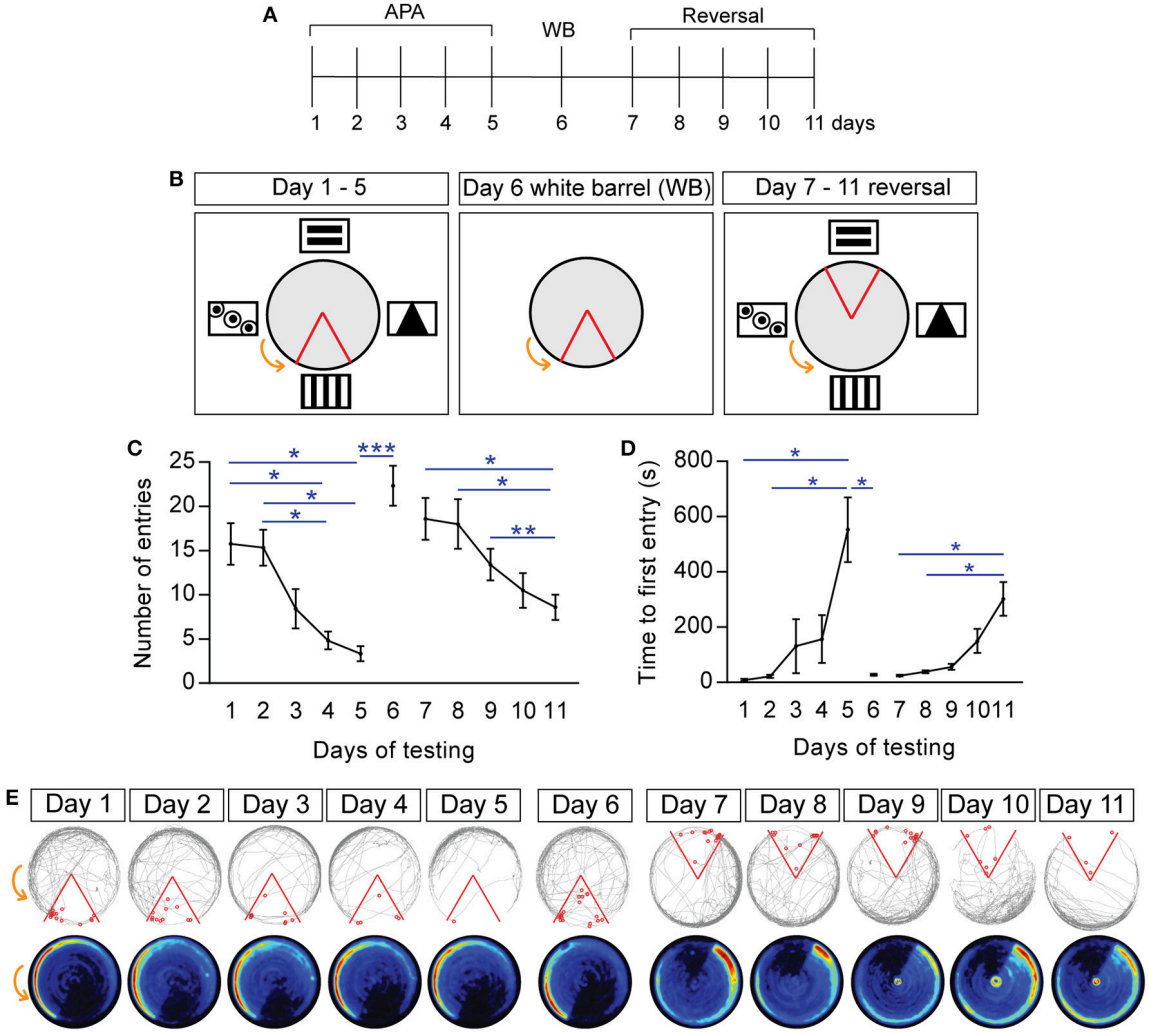
Spatial learning and memory in active place avoidance task is allothetic, with mice able to learn reversed shock zone location. **(A)** Schematic of experimental design and specific testing days. **(B)** Schematic of the shock zone location (red boundary) and visual cues during the 5-day paradigm (days 1–5 of testing, *left*), day 6 (“white barrel”, *middle*), and reversal learning (days 7–11, *right*). **(C)** Number of entries into the shock zone during the 20-min trial over days 1–11 of the testing paradigm, measuring cumulative performance in the task; note that removal of visual cues (“white barrel”; day 6) impairs performance, as does changing the shock zone location (day 7–11). **(D)** Latency, i.e., time to first entry into the shock zone. **(E)** Examples of tracks and mean heat maps of mice during days 1–5, “white barrel” (day 6), and reversal learning (days 7–11). The gray line represents the path of a single mouse, with entry into the shock zone (red boundary) eliciting a shock (red circles). Heat maps represent the merged maps of all mice, where the color of a pixel represents the average trajectory of a track at that location (blue = low, red = high proportion of time; *n* = 12). All data is represented as mean ± SEM; ^*^P < 0.05, ^**^P < 0.01, ^***^P < 0.001, *n* = 12.

To assess spatial memory, mice were retested in the APA over five consecutive days, with each trial 24 h apart (Figure 2A). During days 1–5 of training, the location of the shock zone and the spatial cues remained constant (Figure 2B, left). This spatial learning and memory is evidenced by mice entering into the shock zone significantly less often on days 4 and 5 of testing than on day 1 (day 4: 4.83 ± 1.01 vs. day 1: 15.75 ± 2.36, *P* = 0.032; day 5: 3.33 ± 0.85 vs. day 1: 15.75 ± 2.36, *P* = 0.026; *n* = 12; Figure 2C). This decline in number of entries is closely reflected by the number of shocks received, with fewer shocks on days 4 and 5 of testing than on day 1 (day 4: 5.08 ± 1.05 vs. day 1: 16.67 ± 2.39, *P* = 0.018; day 5: 3.33 ± 0.85 vs. day 1: 16.67 ± 2.39, *P* = 0.014; *n* = 12). There was no significant difference in terms of the distance traveled on days 1–5 of testing (day 1: 50.72 ± 1.27 m; day 5: 48.26 ± 1.88 m; *n* = 12). Mice were also observed to first enter the shock zone significantly later on day 5 of testing than on day 1 and day 2 (Figure 2D; day 2 vs. day 1: 8.81 ± 4.18 s vs. 22.3 ± 5.48 s; day 5 vs. day 1: 552.7 ± 116.6 s vs. 8.81 ± 4.18 s, *P* = 0.041; day 5 vs. day 2: 552.7 ± 116.6 s vs. 22.3 ± 5.48 s, *P* = 0.049; *n* = 12). Similar to the 1-day learning paradigm, spatial learning and place specific avoidance of the shock zone is evidenced by the representative tracked path and heat maps of the mean proportion of time spent at that location (*n* = 12 merged heat maps per day; Figure 2E).

The spatial learning and memory observed during the APA task is believed to be allothetic, i.e., based on the extra-maze visual cues placed around the arena. To confirm this for our testing arena, we removed the visual cues and replaced the clear arena barrier with an opaque white boundary (“white barrel”; Figure 2B, middle), followed by re-testing the mice in a single 20-min APA task. During the white barrel trial, removal of the spatial cues with which the mice would otherwise use to guide themselves and actively move away from the shock zone, results in the mice being passively dragged into the shock zone. The mice were observed to perform poorly in the “white barrel” trial (day 6), entering the shock zone significantly more often compared to day 5 of testing, as well as receiving significantly more shocks compared to day 5 of testing (Figure 2C; number of entries, day 5: 3.33 ± 0.85 vs. day 6: 22.33 ± 2.25, *P* = 0.0001; number of shocks, day 5: 3.33 ± 0.85 vs. day 6: 23.92 ± 1.87, *P* < 0.0001). The time to first entry into the shock zone was also significantly sooner for the “white barrel” trial compared with day 5 of training (day 5: 552.7 ± 116.6 s vs. “white barrel”: 27.87 ± 3.05 s; *P* = 0.048; Figure 2D). Entry into the shock zone during the white barrel trial still elicits an escape response, resulting in the apparent preference for the left quadrant (Figure 2E). Additionally, it is possible that the animals may use non-spatial strategies to avoid shock, walking counter-rotation, evident by the heatmaps for day 6 compared with day 5, however a purely procedural locomotor strategy is insufficient for efficient performance in the task as evident by no significant differences in terms of number of entries into the shock zone during the different time intervals (0–5 min: 5.5 ± 1.31 vs. 15–20 min: 2.75 ± 0.63; *P* = 0.25), or the number of shocks received (0–5 min: 6 ± 1.31 vs. 15–20 min: 2.75 ± 0.63; *P* = 0.15). No significant differences in terms of the distance traveled were observed in the “white barrel” trial, including between the different time intervals (0–5 min: 11.38 ± 0.39 m vs. 15–20 min: 10.70 ± 1.07 m).

To assess whether the mice could learn a new shock location, we conducted reversal learning. In reversal learning, the shock zone is located 180° opposite the original location, with the same visual cues as those used in the 5-day paradigm (day 7–11; Figure 2B, right). Mice were observed to learn the location of the new shock zone, with significantly less entries into the shock zone on days 11 of testing compared with day 7 and 8 (day 7 vs. day 11: 18.58 ± 2.36 vs. 8.58 ± 1.44, *P* = 0.026; day 8 vs. day 11: 18 ± 2.81 vs. 8.58 ± 1.44, *P* = 0.049; day 9 vs. day 11, *P* = 0.006; Figure 2C). The challenging nature of the reversal learning task is evidenced by the mice entering the shock zone significantly more on day 7 compared with day 5 of training (day 5: 3.33 ± 0.85 vs. day 7: 18.58 ± 2.36, *P* = 0.0011). The mice also entered the shock zone significantly sooner on day 7 compared with day 5 of training (day 5: 552.7± 116.6 s vs. day 7: 24.75 ± 3.15 s, *P* = 0.048; Figure 2D). There was no significant difference in terms of number of entries on day 5 vs. day 11 of testing (*P* = 0.40). Mice also entered the shock zone significantly later on day 11 compared with day 7 and 8 of reversal learning (day 7: 24.75 ± 3.15 s vs. day 11: 301.9 ± 61.03 s, *P* = 0.044; day 8: 38.86 ± 4.97 s vs. day 11: 301.9 ± 3.15 s, *P* = 0.046). There was no difference in terms of the distance traveled during reversal learning (day 7: 52.50 ± 2.46 m, day 11: 50.72 ± 1.27 m; *n* = 12).

## Discussion

The APA task is a conditioned behavior that mice can rapidly learn and remember, enabling examination of spatial learning acquisition over a single 20-min learning event (i.e., short term memory). Efficient acquisition of spatial learning in the APA task requires the animal to identify and use the allothetic room-based extra-maze visual cues that are stable with the shock zone to identify and then avoid the shock zone location (Fajnerova et al., 2014). This is demonstrated by the “white barrel” trial, with the rotation of the arena making the substratal idiothetic arena-based coordinate frame unstable (Cimadevilla et al., 2001a,b; Stuchlik et al., 2001). During the APA task, the mice were observed to use two main learning strategies to avoid the shock zone: (1) staying directly opposite the shock zone; or, (2) staying 45° away from the shock zone. Both strategies involve recognition of the shock zone location, and periodically moving in the direction opposite to the counter-clockwise rotation of the arena. On occasion, we have also observed that mice may also use the strategy of sitting in the center of the arena to avoid entry into the shock zone. Maladaptive strategies include freezing (suppression of locomotion) following escape from the shock zone, as the arena rotation will carry the animal back into the shock zone (Cimadevilla et al., 2001b). Additionally, use of olfactory cues (substratal exteroceptive cues) to define and learn the location of the shock zone is eliminated by to the constant rotation of the arena, with the shock zone defined solely by its room frame coordinates. Other strategies, such as a locomotor or procedural strategies, in which the animals simply walk at a constant velocity counter-rotation without any spatial awareness, may be employed, however they are not sufficient for efficient performance in the APA task, as evident during the “white barrel” trial. In line, naïve rats unable to learn the shock zone location in the dark, with only rats familiar with the task and the task rules able to perform the task, however in the dark (with absence of visual cues) even performance of rats familiar with the task was impaired compared to performance in the light (Stuchlik et al., 2013). In the protocol described herein, young wild-type mice were observed to rapidly learn the spatial relationship between the visual cues and the shock zone during the 1- and 5-day learning paradigms, as well as the reversal learning paradigm. In addition, the learning curve, as described for the 1-day learning paradigm, can be extracted for any of the 20-min trials during the 5-day learning paradigm as well as the reversal learning paradigm.

Reversal learning, also termed proactive interference learning, is a useful method for increasing the cognitive load and assessing subtle changes in spatial cognition (Epp et al., 2016). Adjustment to the new shock zone location requires a specific kind of cognitive flexibility, previously described as mnemonic segregation; that is, the ability to segregate a relevant novel experience (the reversed shock zone location) from a previously established memory (the initial shock zone location) (Abdel Baki et al., 2009). During reversal learning, the old and irrelevant spatial representation must be distinguished from the new and relevant spatial representation, while the need to segregate spatial frames (arena- and room-based) remains (Kelemen and Fenton, 2010). The ability to differentiate between similar memories and choose the appropriate behavior is dependent on hippocampal pattern separation (Burghardt et al., 2012). This cognitive flexibility is also reliant on the dentate gyrus, demonstrated to play an important role in dissociating between contextual encoding of spatial memories and discriminating between conflicting memories (Kheirbek et al., 2013). In addition, hippocampal neurogenesis is important for interference learning, as demonstrated by increased neurogenesis specifically facilitating high interference learning (i.e., encoding of new, conflicting information in mice), but not generally improving paired associate learning (Epp et al., 2016). Moreover, genetic suppression of hippocampal neurogenesis by valganciclovir treatment in nestin-thymidine kinase mice impairs high interference learning, but not low interference leaning (Epp et al., 2016).

We and others have also used the APA task to investigate the role of hippocampal neurogenesis in spatial learning and memory more generally (Sahay et al., 2011; Burghardt et al., 2012; Vukovic et al., 2013; Park et al., 2015; Cleland et al., 2017). We demonstrated that pharmacogenetic ablation of immature doublecortin-positive hippocampal neurons impairs performance in a novel, but not in a familiar, spatial learning task (Vukovic et al., 2013). This highlights the importance of immature hippocampal neurons in the acquisition of spatial learning but not retrieval of long-term spatial memory (Vukovic et al., 2013). Additionally, we have used the 1-day paradigm to assess spatial learning deficits in 18-month old wild-type mice (Cleland et al., 2017). As the protocol described herein can be utilized to assess short term memory (1-day paradigm) as well as longer term memory (5-day paradigm) the importance of hippocampal neurogenesis for such cognitive functions can now be addressed in future studies. Longer term memory assessed by the APA task has also been demonstrated by others, whereby extensively training mice in the APA task results in long-term persistent modifications in CA1 hippocampal circuitry, as demonstrated by increased synaptic transmission of the evoked field excitatory postsynaptic response and decreased potentiation of the CA3-CA1 input 30 days after the final training session (Pavlowsky et al., 2016).

### Significance of the technique with respect to existing methods

The APA task offers many methodological advantages over other approaches, with the greatest advantage being the very rapid acquisition of spatial learning, as this enables assessment of short-term changes in performance. This is demonstrated in the current paradigm, where mice learnt the position of the shock zone during a single 20-min spatial learning event. The APA task is a more sensitive measure of hippocampal impairment than the widely used Morris water maze (Cimadevilla et al., 2000a). This was demonstrated by unilateral hippocampal inactivation with tetrodotoxin injections not impairing performance in the water maze variable-start, fixed-hidden goal task, whereas the same mice injected with tetrodotoxin performed significantly worse in the APA task compared with controls (Cimadevilla et al., 2001a,b; Kubik and Fenton, 2005). Hence, the APA task is suggested to require greater hippocampal integrity than the Morris water maze task (Cimadevilla et al., 2001b). The allothetic APA task is also learnt faster than the Morris water maze and, unlike the water maze, both mice and rats appear to learn the APA task at the same rate (Cimadevilla et al., 2001a,b).

Future studies can use the APA task as a robust and effective measure for evaluating experimental interventions (such as brain lesions or pharmacological interventions) on cognitive aspects of spatial behavior, or investigations of animal models of psychiatric disorders and brain injuries, or unit electrode recordings from behaving animals (Stuchlik et al., 2013). Indeed, one such study already demonstrated that repeated ultrasound scanning treatments to remove amyloid-β from the brains of APP23 Alzheimer's disease transgenic mice improves performance in the APA task, highlighting its potential as a non-invasive therapeutic intervention to improve cognition (Leinenga and Gotz, 2015).

### Critical steps and troubleshooting

One of the most critical steps in this protocol is ensuring that the mice are properly habituated to both the experimenter and the testing arena. Poor performance, evidenced by no change in the number of entries or number of shocks received during the different time intervals of the trial, may be indicative of anxiety-related freezing behavior, which prevents efficient learning (Carr et al., 2011; Stuchlik et al., 2013). In the current protocol, we did not observe any anxiety-related behaviors such as jumping or freezing. Additionally, we have never observed a mouse jumping over the 32-cm high arena boundary; however, if a mouse is capable of jumping over, further testing for that mouse should be terminated. Attention should be given to the foot shock current, as intensity—both too low and too high—can compromise the animal's motivation or ability to learn and perform the task (Stuchlik et al., 2013). Foot shocks at a lower intensity than that used in this protocol (0.5 mA) may be used, with 0.2 mA reported to be the minimum necessary to elicit flinch or escape responses in mice Performance in the APA task may also be improved by either making the two-dimensional cues more obvious or using three-dimensional visual cues, so long as these cues remain stable during the trial.

## Limitations of the protocol

The APA task is a rather complex and challenging task, measuring subtle changes in spatial learning. Interventions (genetic or pharmacological) that may impede the ability of the mice to perform the test, such as those that impair their locomotor abilities, and hence impair the mouse's ability to escape from the shock zone, may not be appropriate to test in this task. It should also be noted that we have found the APA task to be more suitable for adult and aged mice; juvenile mice (<8 weeks old) tend to use the maladaptive strategy of trying to jump out of the testing arena in order to avoid being shocked (*unpublished observations*). Long-term spatial memory assessed in this protocol is established after four learning days, which may present as a limitation for interventions that may only act for a short (>4 day) period; however, for such interventions the impact on working memory, short term memory, and acquisition of spatial learning can still be assessed using the current protocol.

## Author contributions

JV and PB designed the project. EW performed the experiments and analyzed the results. EW and JV wrote and reviewed the manuscript. All authors approved the manuscript.

### Conflict of interest statement

The authors declare that the research was conducted in the absence of any commercial or financial relationships that could be construed as a potential conflict of interest.
